# Small for gestational age and early childhood caries: the BRISA cohort study

**DOI:** 10.1038/s41598-023-41411-y

**Published:** 2023-09-01

**Authors:** Juliana de Kássia Braga Fernandes, Francenilde Silva de Sousa, Cláudia Maria Coelho Alves, Cecília Cláudia Costa Ribeiro, Vanda Maria Ferreira Simões, Maria da Conceição Pereira Saraiva, Erika Barbara Abreu Fonseca Thomaz

**Affiliations:** 1 Programa de Pós-graduação em Saúde Coletiva, Universidade Federal do Maranhão, São Luís, MA Brazil; 2 Programa de Pós-graduação em Odontologia, Universidade Federal do Maranhão, São Luís, MA Brazil; 3 Faculdade de Odontologia de Ribeirão Preto, Universidade São Paulo, São Paulo, SP Brazil; 4 Departamento de Odontologia, Universidade CEUMA, São Luís, MA Brazil; 5 Departamento de Saúde Pública, Universidade Federal do Maranhão (UFMA), Rua Barão de Itapari, 155 – Centro, São Luís, Maranhão 65020-070 Brazil

**Keywords:** Risk factors, Dental diseases, Oral diseases

## Abstract

This study tests the hypothesis that children 12–30 months born small for gestational age (SGA) aged are more susceptible to severe early childhood caries (S-ECC). We used data on 865 children aged 12–30 months from a prospective cohort study conducted in a city in the northeast of Brazil. The study outcome was S-ECC, defined based on the proportion of decayed tooth surfaces (cavitated or not). The main exposure variable was SGA, defined according to the Kramer criterion and the INTERGROWTH-21st standard. Direct (SGA → S-ECC) and indirect effects were estimated using structural equation modeling, calculating standardized factor loadings (SFL) and P-values (alpha = 5%). The final models showed a good fit. SGA influenced S-ECC in the direct and indirect paths. In the group of SGA children with 12 or more erupted teeth defined according to the Kramer criterion, the direct effect was positive (SFL = 0.163; P = 0.019); while among all SGA children defined according to the INTERGROWTH-21st standard, the direct effect was negative (SFL =  − 0.711; P < 0.001). Age and number of erupted teeth may influence the occurrence of S-ECC in SGA children, as the number of teeth affects the time of exposure to disease risk factors.

## Introduction

Early childhood caries (ECC) is a chronic disease that affects one or more primary teeth in children aged up to 72 months (cavitated or non-cavitated lesions, teeth lost due to caries, or restored teeth). ECC remains a significant public health problem. In children under the age of three, the disease is called severe early childhood caries (S-ECC)^1^. Studies show that ECC has a major impact on the quality of life of children and their families^2^.

Adverse birth events are associated with an increased risk of both ECC^2^ and S-ECC^3^. One such event is small for gestational age (SGA), where birthweight is either 15% lower than the 50th percentile line of the birthweight for gestational age curve or below the INTERGROWTH-21st 10th percentile. In low- and middle-income countries, 27% of live births are small-for-gestational-age^4^. The relationship between SGA and S-ECC may be mediated by the higher incidence of developmental defects of enamel (DDE) in the primary dentition of infants^5, 6^.

The hypothesis that SGA children are more likely to develop DDE is supported by evidence suggesting that primary teeth with DDE may have a more irregular surface, which facilitates bacterial adhesion and colonization, predisposing the individual to caries^5, 6^. There is also evidence that SGA children may be immunodeficient, increasing the incidence of infections during the first few months of life^6^. Low immunity can affect oral flora, leading to an increase in the number of *Streptococcus mutans*^7^ and favoring the formation of caries, especially in more susceptible substrates such as primary teeth with DDE.

Not all children with *S. mutans* develop caries, even when they have similar oral hygiene, diet, and environmental characteristics. This suggests that host susceptibility potentially plays a role in the development of dental caries; however, the association between host genetics, *S. mutans*, and dental caries remains unclear^8, 9^. A systematic review published in 2019^10^ concluded that both DDE and high levels of *S. mutans* were strong risk factors for S-ECC. In addition, the cariogenic potential of *S. mutans* strains depends on the presence of clinical serotypes^11^.

Studies investigating the relationship between adverse birth events and caries have tended to focus on children aged over two^12^. However, the effect of these events on caries development may be less evident in older infants because it is masked by other factors such as sugar consumption and bad oral hygiene habits, making it more difficult to identify the association between events and S-ECC.

Another relevant point concerns the method used to diagnose ECC. Most studies use the DMFT (decayed, missing, and filled teeth) index, which does not assess caries activity. This method does not take into account white spots, which can be an early sign of tooth decay, meaning that it may underestimate caries activity, especially in younger children^13^.

This study assesses the biological plausibility of the association between SGA and S-ECC and seeks to identify gaps in the literature on this topic, especially regarding the relationship between adverse birth events and S-ECC. To this end, we used structural equation modeling (SEM) to test the hypothesis that children 12–30 months born SGA aged are more susceptible to S-ECC. We also investigated whether disease activity was mediated by the presence of DDE.

## Methods

### Study design and location

We carried out a prospective cohort study using data from the BRISA Cohort Study, conducted in two cities in Brazil: São Luís (MA) and Ribeirão Preto (SP)^14^. The two cities are located in different regions of the country and display widely varying cultural, socioeconomic, and demographic characteristics. So, the present study focused on only one of the cities (São Luís) to provide a more homogeneous dataset and minimize confounding bias. Besides, In addition, there were no data for some important variables in these studies collected in a standardized way in both municipalities. The study was conducted following the guidelines of the STROBE Statement.

São Luís is the capital of Maranhão, a state in the northeast of Brazil with one of the lowest human development indices (0.639) in the country. The estimated population of the city in 2010 was 1,014,837^15^. The average annual number of live births between 2010 and 2013 was 21,428^16^. A study carried out using data from 2009 showed that while many of the 480 water samples had optimal fluoride levels, access to fluoridated water was unequal, with poorer areas having lower levels of fluoride^17^.

The data were collected at three separate intervals between January 2010 and March 2013: during antenatal care (T1); first follow-up at birth (T2); and second follow-up at age 12–30 months (T3).

### Sample

The study sample consisted of liveborn singleton children who participated in the baseline study, were followed up at T2 and T3, and underwent a dental examination (n = 865) and their mothers.

The sample of 865 children was estimated to have a power of 95.76% to detect associations between SGA and S-ECC, calculated based on the following parameters: alpha = 0.05 (bilateral); mean (± standard deviation) number of carious surfaces equal to 0.40 (± 1.54) in the exposed group and 1.69 (± 7.63) in the unexposed group; sample of 46 exposed and 819 unexposed individuals.

The study sample was selected using convenience sampling because it was not possible to obtain a representative random sample of pregnant women since there is not a reliable record of pregnant women and/or women receiving antenatal care in the state of Maranhão. Available data is neither updated nor validated. So, the women were selected during visits to public and private health centers for antenatal/follow-up care. Inclusion criteria included having performed an ultrasound scan before 20 weeks of gestation and not having completed more than 25 weeks of gestation at the time of baseline data collection. The baseline data were collected at the Clinical Research Center (CEPEC) between January 2010 and June 2011.

### Data collection procedures

The data were collected using clinical examinations and three questionnaires: the mother’s questionnaire at T1, including questions on socioeconomic and demographic characteristics; the newborn questionnaire at T2, containing questions on weight and gestational age at birth; and the child questionnaire at T3, to obtain data on age, diet, and consumption of sugar-sweetened foods.

A dental examination was performed at T3 to assess DDE, S-ECC, VPI (used as a proxy measure of oral hygiene), and the number of teeth. At the end of the examination, the dentist brushed the child’s teeth to check for bleeding gums during brushing. Six examiners were trained to perform the examination (inter- and intra-rater Kappa ≥ 0.80). The data were recorded on a specific individual form for each child. The dental examinations were performed at the Maternal and Infant Health Unit (HUUMI) of the Federal University of Maranhão University Hospital (HUUFMA) following World Health Organization (WHO) recommendations: under artificial lighting, using mouth mirrors, periodontal probes, compressed air, distilled water, and a three-way syringe^18^.

### Variables

The study outcome was S-ECC, characterized by the ratio between the number of decayed tooth surfaces and the total number of tooth surfaces. The presence of the disease was determined using the Nyvad codes and criteria: 1—Active caries without surface discontinuity (intact surface); 2—active caries (microcavity); 3—active caries (cavity); 5—inactive caries (microcavity); and 6—inactive caries (cavity)^19^. Nyvad is a validated classification system^19^, has been shown to have high reproducibility for lesion severity assessment when compared to the International System for the Detection and Evaluation of Caries (ICDAS)^20^, and has been used in international studies^21, 22^.

The main independent variable was SGA^23^, determined based on Kramer et al.^24^ and the INTERGROWTH-21st standard^25^. According to Kramer, SGA is defined by dividing birthweight (in g) by the weight on the 50th percentile line of the birthweight for gestational age curve based on the Canadian birthweight standards^24^.

According to the INTERGROWTH-21st standard, SGA babies have birthweight below the 10th percentile, meaning they are smaller than 90% of (most) other babies of the same gestational age. The INTERGROWTH-21st standards were produced using data from a multicenter multiethnic population-based project conducted between 2009 and 2014 in eight developed and developing countries, including Brazil. The primary aim of the project was to study growth, health, nutrition, and neurodevelopment from 14 weeks of gestation to 2 years of age^25^.

The potential mediating variables tested by this study were DDE, number of erupted teeth, and visible plaque index (VPI). DDE was diagnosed using the Fédération Dentaire Internationale Defects of Enamel index (1982)^26^, which categorizes DDE as diffuse opacities, demarcated opacities, and hypoplasia. Tooth surfaces with any of the three types of DDE were counted. The analysis considered the total number of teeth present in the oral cavity. The tooth was considered present in the oral cavity when any part of the tooth was visible during the examination. The VPI was also assessed during the dental examination.

The confounding variables were socioeconomic status, oral hygiene, and eating habits. Socioeconomic status was a latent variable measured using the following variables: family income (in minimum wages for the baseline year 2010); occupation of the head of the family (from unskilled job to manager/business owner); economic classification based on the criteria proposed by the Brazilian Market Research Association (ABEP)—(A/B; C; or D/E)^27^; and maternal education (≤ 4 to ≥ 12 years). These variables are important indicators of caries^28^. Oral hygiene and eating habits were assessed by asking the child’s mother/caregiver the following questions: Do you brush your child’s teeth every night before they go to bed? (yes/no); Does your child eat sugar-sweetened foods? (yes/no).

These variables were chosen based on the assumption that teeth with enamel defects are likely to have higher levels of cariogenic biofilm formation (measured by the VPI) when fermentable sugars are consumed, increasing the incidence of S-ECC. The consumption of sugar-sweetened foods was chosen as a measure of eating habits because sugar is the main cause of caries^29^. The VPI was used because this measure indicates severe chronic plaque buildup, which, in the presence of sugar, can lead to caries^30^. Nighttime brushing was chosen as a measure of oral hygiene habits because evidence suggests that this method is more effective for controlling cariogenic activity than daytime brushing^10^.

### Statistical analysis

**Figure 1:**
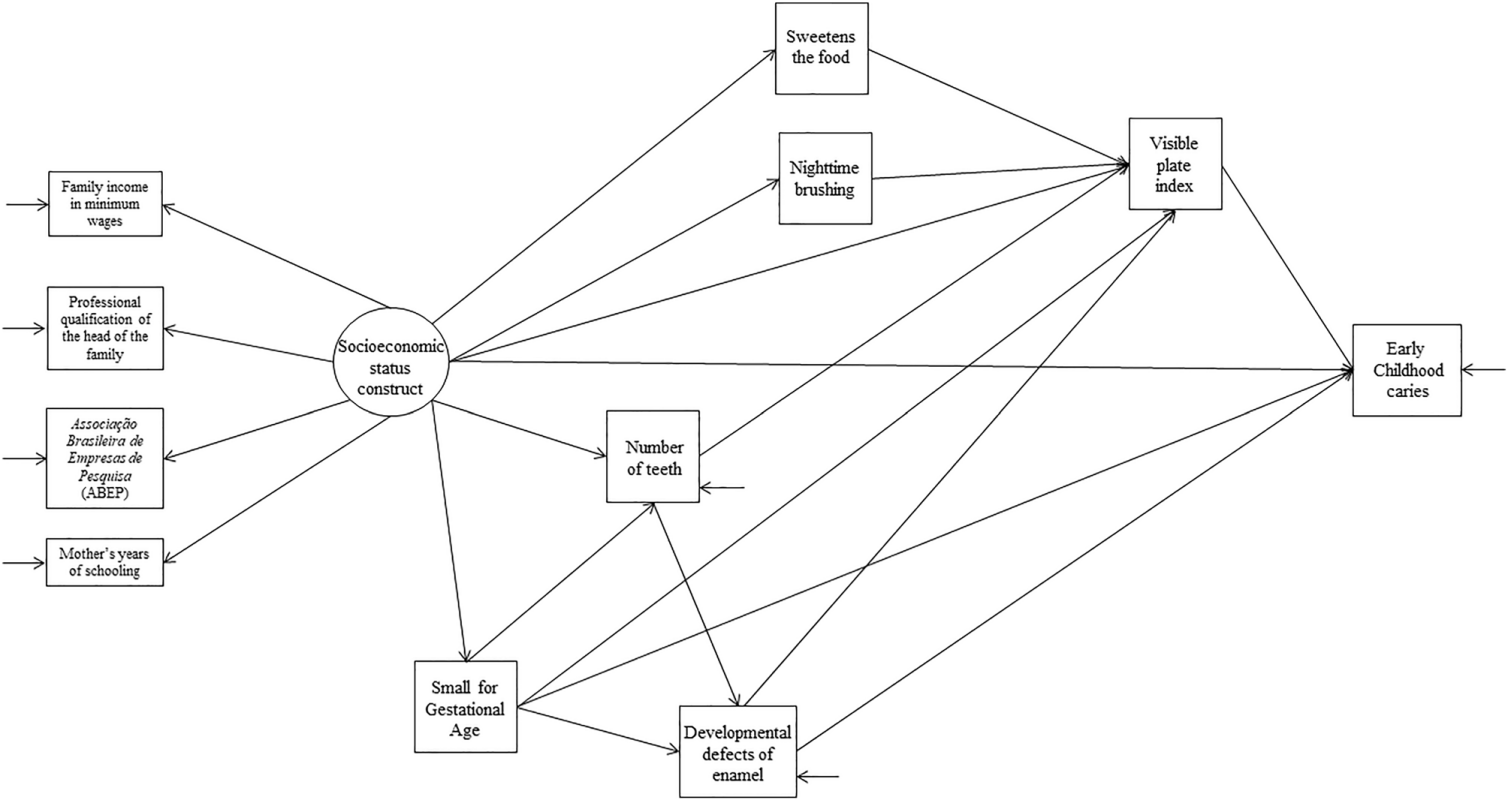
Structural equation modeling of the hypothesis (SGA increases the risk of DDE-mediated S-ECC).

The association between SGA, DDE, and other covariates and S-ECC was assessed using a theoretical model (Fig. 1) tested using SEM. The statistical analyses were performed using Stata/SE 12.0 and Mplus 7.3 software. Absolute and relative frequencies were presented for the three study intervals to identify follow-up losses. Differences were estimated using chi-squared or Fisher’s exact test.

The latent variable was modeled using variable indicators. A good latent variable should have adequate convergent validity, showing that the indicator correlates with other indicators used to measure the same construct. Standardized factor loadings (SFL) greater than 0.60 indicate adequate convergent validity. A latent variable must also have adequate discriminant validity, when correlations between indicators are not excessively high (> 0.95), showing that each indicator measures different aspects of the construct. Negative loadings indicate an inverse association and positive loadings indicate a direct association^31^.

We adopted the SFL thresholds proposed by Kline^31^. Coefficients with values close to 0.10 indicate a small effect; around 0.30, a medium effect; and greater than 0.50, a strong effect. The tested models were evaluated using fit indices. The following values were considered acceptable: RMSEA (root mean square error of approximation) < 0.05; CFI (comparative fit index) and TLI (Tucker–Lewis index) > 0.95; and WRMR (weighted root mean square residual) < 0.95. Chi-squared, degrees of freedom, and P-values were calculated, but were not adopted as parameters to determine model fit due to the large sample size, which could influence the results of these tests.

The models were evaluated using mean- and variance-adjusted weighted least squares (WLSMV), indicated for the analysis of categorical data^31^. Theta parameterization was used to control for differences in residual variance. The automatic command “MODINDICES” was used to suggest modifications to the initial model. When the proposed modifications are considered theoretically plausible and the modification index is greater than 10,000, a new model can be developed and analyzed.

### Ethical considerations

This study protocol was approved by the HUUFMA Research Ethics Committee (Reference Code 4771/2008-30). All mothers signed an informed consent form explaining the nature of the study. Thus, all methods were performed following relevant guidelines and regulations.

## Results

The incidence of SGA based on the Kramer criterion and INTERGROWTH-21st standard was 18.18% (n = 251) and 2.97% (n = 41), respectively. The incidence of gum bleeding during brushing was 6.71% (n = 58), and 12.26% (n = 106) of the children were diagnosed with some type of EDD. The incidence of S-ECC was 5.20% (n = 45), with a mean of 0.37% (± 2.39%) of decayed tooth surfaces. The average number of teeth per child at the time of the examination was 9.75 (± 3.75) (Table 1). The ratios for the variables in Table 1 remained similar between T1, T2, and T3, indicating that sample losses to follow-up were non-differential.

**Table 1:** Absolute and relative frequencies of study variables. São Luís/MA, Brazil, 2010-2013.

Variable	Antenatal T1 (n = 1447)		Birth T2 (n = 1381)		12–30 months T3 (n = 1151)		Dental examination T3 (n = 865)	
	n^a^	%^b^	n^a^	%^b^	n^a^	%^b^	n^a^	%^b^
Family income in minimum wages								
< 1 minimum wage	17	1.17	17	1.23	12	1.04	9	1.04
Between 1 and 3 minimum wages	669	46.23	638	46.20	533	46.31	413	47.75
Between 3 and 5 minimum wages	448	30.96	428	30.99	365	31.71	280	32.37
≥ 5 minimum wages	270	18.66	258	18.68	209	18.16	139	16.07
Information not available	43	2.97	40	2.90	32	2.78	24	2.77
Occupation								
Unskilled job	396	27.37	379	27.44	310	26.93	230	26.59
Nonspecialized manual job	565	39.05	540	39.10	465	40.40	351	40.58
Specialized manual job	66	4.56	65	4.71	51	4.43	41	4.74
Office job	218	15.07	205	14.84	166	14.42	131	15.14
The job that requires a degree	77	5.32	69	5.00	59	5.13	43	4.97
Managerial position/business owner	43	2.97	42	3.04	38	3.30	26	3.01
Information not available	82	5.67	81	5.87	62	5.39	43	4.97
Economic classification—ABEP								
D/E	226	15.62	213	15.42	173	15.03	125	14.45
C	933	64.48	893	64.66	753	65.42	579	66.94
A/B	221	15.27	211	15.28	173	15.03	123	14.22
Information not available	67	4.63	64	4.63	52	4.52	38	4.39
Maternal schooling								
≤ 4 years	23	1.59	23	1.67	17	1.48	12	1.39
5 to 8 years	146	10.09	146	10.57	115	9.99	92	10.64
9 to 11 years	1057	73.05	1057	76.54	886	76.98	668	77.23
≥ 12 years	154	10.64	154	11.15	129	11.21	90	10.40
Information not available	67	4.63	1	0.07	4	0.35	3	0.35
Birthweight								
> 2500 g			1272	92.11	1076	93.48	809	93.53
1500 to 2500 g			86	6.23	61	5.30	44	5.09
< 1500 g			11	0.80	5	0.43	4	0.46
Information not available			12	0.87	9	0.78	8	0.92
SGA (Kramer criterion)								
No			1116	80.81	940	81.67	714	82.55
Yes			251	18.18	202	17.55	143	16.53
Information not available			14	1.01	9	0.78	8	0.92
SGA (INTERGROWTH-21st standard)								
No			1325	95.94	1113	96.70	836	96.65
Yes			41	2.97	27	2.34	21	2.43
Information not available			15	1.09	11	0.96	8	0.92
Consumption of sugar-sweetened foods								
No					254	22.07	210	24.28
Yes					892	77.50	642	74.22
Information not available					5	0.43	13	1.50
Nighttime brushing								
No					394	34.23	292	33.76
Sometimes					351	30.50	268	30.98
Always					404	35.10	294	33.99
Information not available					2	0.17	11	1.27
DDE								
No							751	86.82
Yes							106	12.26
Information not available							8	0.92
Presence of caries								
No							810	93.64
Yes							45	5.20
Information not available							10	1.16

							Mean (sd^c^)	Min–max
% of decayed tooth surfaces							0.37 (2.39)	0–33.33
Number of teeth							9.75 (3.75)	0–20

*T1* Time 1 (Baseline), *T2* Time 2 (1st follow-up), *T3* Time 3 (2nd follow-up), *min–max* minimum–maximum, *ABEP Associação Brasileira de Empresas de Pesquisa* (the Brazilian Market Research Association), *SGA* small for gestational age, *DDE* developmental defects of Enamel.

^a^Absolute frequency.

^b^Relative frequency.

^c^Standard deviation (sd).

^1^Baseline value for a minimum wage in 2010 = R$510.00.

*Excluded values ignored or not informed.

The Supplementary Material presents the prevalence ratios for S-ECC by age (months), showing that the prevalence of caries increases with the increasing number of teeth.

The final models including all SGA children-based on the Kramer criterion (RMSEA = 0.024; CI 0.014–0.033; CFI = 0.961; TLI = 0.940; and WRMR = 0.846) and the INTERGROWTH-21st standard (RMSEA = 0.023, CI 0.013–0.033, CFI = 0.949, TLI = 0.916 and WRMR = 0.713)—showed good fit. The final models including SGA children with 12 or more teeth also showed a good fit (Table 2).

**Table 2:** Fit indices for the final model. São Luís/MA. Brazil, 2023.

Index	Kramer criterion		Intergrowth-21st standard		Reference value
	All SGA children (n = 865)	SGA children with 12 or more teeth (n = 289)	All SGA children (n = 865)	SGA children with 12 or more teeth (n = 289)	
	Value				
X^2a^	62.639	35.371	58.424	45.818	–
Degrees of freedom	34	34	33	33	–
P-value	0.0020	0.4033	0.0041	0.0681	> 0.05
RMSEA	0.024	0.012	0.023	0.029	< 0.05
90% CI	0.014–0.033	0.000–0.045	0.013–0.033	0.000–0.047	< 0.08
CFI	0.961	0.988	0.977	0.949	> 0.95
TLI	0.940	0.981	0.962	0.916	> 0.95
WRMR	0.846	0.615	0.812	0.713	< 0.95

*SGA* small for gestational age, *RMSEA* root mean square error of approximation, *CI* confidence interval, *CFI* comparative fit index, *TLI* Tucker Lewis index, *WRMR* weighted root mean square residual.

^a^Chi-squared test.

The latent variable socioeconomic status showed a high SFL (around 0.50) across all variables. SGA affected S-ECC in both the direct and indirect paths. In the group of SGA children with 12 or more teeth defined according to the Kramer criterion, the direct effect was positive (SFL = 0.163; P = 0.019), while in the group defined according to the INTERGROWTH-21st standard, the direct effect was negative (SFL =  − 0.711; P < 0.001) (Table 3).

**Table 3:** Factor loading and P-values to assess construct validity and model factor structure. São Luís/MA. Brazil, 2023.

	Kramer criterion				INTERGROWTH-21st standard			
	All SGA children (n = 865)		SGA children with 12 or more teeth (n = 289)		All SGA children (n = 865)		SGA children with 12 or more teeth (n = 289)	
	SFL^a^	P^b^	SFL^a^	P^b^	SFL^a^	P^b^	SFL^a^	P^b^
Construct								
SES^1^								
A^2^	**0.636**	** < 0.001**	**0.618**	** < 0.001**	**0.635**	** < 0.001**	**0.640**	** < 0.001**
B^3^	**0.525**	** < 0.001**	**0.535**	** < 0.001**	**0.526**	** < 0.001**	**0.579**	** < 0.001**
C^4^	**0.793**	** < 0.001**	**0.827**	** < 0.001**	**0.796**	** < 0.001**	**0.812**	** < 0.001**
D^5^	**0.428**	** < 0.001**	**0.338**	** < 0.001**	**0.429**	** < 0.001**	**0.448**	** < 0.001**
Direct paths								
SGA → S-ECC	− 0.061	0.143	**0.163**	**0.019**	− **0.711**	** < 0.001**	0.043	0.518
SGA → DDE^12^	− 0.024	0.756	0.089	0.258	− 0.044	0.798	0.014	0.898
SGA → VPI	0.034	0.536	− 0.054	0.632	0.065	0.471	0.048	0.675
SGA → NT	− **0.176**	** < 0.001**	**0.175**	**0.034**	− 0.109	0.273	− 0.201	0.058
SES → SGA^6^	− 0.054	0.244	0.056	0.630	− 0.030	0.602	0.074	0.368
SES → SF^8^	**0.094**	**0.020**	0.069	0.405	**0.095**	**0.020**	0.130	0.071
SES → VPI^10^	0.161	0.057	0.085	0.263	− 0.012	0.780	0.093	0.211
SES → NB^7^	− 0.056	0.264	− 0.126	0.215	− 0.056	0.269	− 0.083	0.348
SES → NT^9^	− **0.093**	**0.030**	− 0.024	0.722	− 0.064	0.137	0.013	0.851
SES → S-ECC^11^	− **0.136**	**0.003**	− **0.241**	**0.005**	− **0.136**	**0.023**	− **0.234**	**0.006**
NT → VPI	**0.142**	** < 0.001**	− 0.084	0.164	**0.122**	** < 0.001**	− 0.089	0.162
NT → DDE	**0.036**	**0.048**	0.013	0.884	0.035	0.118	0.030	0.589
DDE → VPI	0.041	0.099	0.032	0.556	0.044	0.112	0.030	0.589
DDE → S-ECC	0.013	0.859	0.009	0.899	− 0.045	0.758	0.000	0.998
NB → VPI	0.030	0.522	− 0.043	0.642	0.029	0.530	− 0.040	0.660
SF → VPI	− 0.018	0.651	− 0.069	0.309	− 0.017	0.655	− 0.082	0.238
VPI → S-ECC	**0.095**	**0.004**	0.058	0.283	0.100	0.172	0.053	0.311
Indirect paths								
SGA → S-ECC								
Total effect	− 0.448	0.133	**0.160**	**0.021**	− **0.705**	** < 0.001**	0.034	0.575
Total indirect effect	0.003	0.950	− 0.004	0.680	0.006	0.771	− 0.009	0.490
Specific indirect effects								
SGA → VPI → S-ECC	0.024	0.550	− 0.003	0.696	0.006	0.627	0.003	0.689
SGA → DDE → S-ECC	− 0.002	0.861	0.000	0.931	0.002	0.885	− 0.012	0.299
SGA → NT → VPI → S-ECC	− **0.017**	**0.047**	− 0.001	0.399	− 0.001	0.456	0.001	0.437
SGA → DDE → VPI → S-ECC	− 0.001	0.773	0.000	0.892	0.000	0.820	0.000	0.898
SGA → NT → DDE → S-ECC	− 0.001	0.864	0.000	0.897	0.000	0.730	0.000	0.998
SGA → NT → DDE → VPI → S-ECC	0.000	0.181	0.000	0.652	0.000	0.455	0.000	0.669

^a^*﻿SFL* standardized factor loading. Reference for factor loading: small effect 0.50.

^b^P-value considered significant: P < 0.05.

^1^*SES* socioeconomic status construct.

^2^A: Family income in minimum wages.

^3^B: Occupation of the head of the family.

^4^C: *Associação Brasileira de Empresas de Pesquisa*—ABEP.

^5^Maternal years of schooling.

^6^Small for gestational age.

^7^Nighttime brushing.

^8^Consumption of sugar-sweetened foods.

^9^Number of teeth.

^10^Visible plaque index.

^11^Early childhood caries.

^12^Developmental defects of enamel.

Significant values are in bold.

SGA defined according to the Kramer criterion had a direct effect on the number of teeth in all SGA children (SFL =  − 0.176; P < 0.001) and in SGA children with 12 or more teeth (SFL = 0.175; P = 0.034) (Table 3).

The number of teeth influenced the VPI in all SGA children based on both the Kramer criterion (SFL = 0.142; P < 0.001) and INTERGROWTH-21st standard (SFL = 0.122; P < 0.001). SGA had a specific negative indirect effect on S-ECC mediated by the number of teeth and VPI (SFL =  − 0.017; P = 0.047). SGA did not have an effect on S-ECC in the other indirect paths tested (Table 3).

## Discussion

Our findings reveal a direct association between SGA and S-ECC, as well as an indirect association mediated by the number of teeth and VPI. The hypothesis that SGA increases the risk of DDE-mediated S-ECC was not supported.

The theoretical basis for this relationship is that nutritional deficiency during pregnancy, which is predicted to be more prevalent in low-income populations^32^ such as the study sample, increases the risk of EDD and, consequently, S-ECC. Studies have suggested a relationship between SGA and EDD mediated by vitamin D deficiency^33–36^. While we did not evaluate nutritional deficiency, considering the socioeconomic characteristics of the study sample, a relationship between SGA and EDD mediated by vitamin D deficiency may be assumed in the present study.

Nonetheless, SGA has multiple causes, including nutritional, genetic, and hormonal factors^37, 38^. Certain factors may lead to intrauterine growth restriction in this population without having a causal effect on EDD, resulting in the absence of an association between SGA and caries.

Age may also be an important factor, as the older the child, the greater the influence of extrinsic variables, which may have a confounding effect on results. The fact that our study sample is very young (up to 30 months) reduces confounding and helps us understand the influence of antenatal factors such as SGA, allowing us to better observe their effects. However, the relationship between adverse birth events and caries, which is a disease that develops over time, may have been underestimated in the study population because, due to the young age of our sample, the disease may not have had time to manifest itself^1^.

The data also point to an indirect association mediated by the number of teeth and VPI: the smaller the children for gestational age, the smaller the number of teeth, the lower the prevalence of VPI, and the lower the incidence of caries at age at the time of examination.

Some authors suggest that adverse perinatal factors such as SGA are possible causal factors for delayed tooth eruption^39^. Furthermore, colonization with *Streptococcus mutans* is determined by the eruption of deciduous teeth and increasing numbers of teeth increase the risk of infection by *S. mutans,* thus increasing the risk of caries^40^.

Thus, SGA may have influenced *S. mutans* colonization, as SGA children had fewer teeth at the time of the examination compared to children with adequate weight for gestational age. Castro et al. found an association between delayed tooth eruption and low prevalence of severe caries^41^, which may partially explain the discrepancies in the associations found between SGA and S-ECC. In this respect, the association was positive in SGA children with 12 or more teeth based on the Kramer criterion and negative in all SGA children defined according to the INTERGROWTH-21st standard.

Neither night brushing nor consumption of sugar-sweetened foods showed a correlation with S-ECC. This may be due to the low variability of this data. Both variables are prone to social desirability bias when respondents tend to provide answers that are more socially acceptable than their true opinions or behavior, leading to data homogeneity and hampering the detection of possible associations. However, our findings do show an association between VPI and S-ECC. The former is an important proxy measure for the presence of mature biofilm and is less susceptible to measurement errors, thus supporting the above explanation^1^.

Our results point to the importance of taking a life-course approach to the treatment of oral disease, as distal variables can play an important role in the causality of the events investigated by this study^42^. Older children with teeth are exposed to risks for longer and therefore more susceptible to oral diseases such as caries^43, 44^.

Although we identified a significant specific indirect path, the main results were derived from estimates of direct effects, which could have been achieved using conventional regression models. However, it is important to note that, conceptually, conventional regression methods are not appropriate for assessing temporal order, since they do not consider the temporal sequence between factors. SEM, on the other hand, addresses these considerations, yet requires a large amount of data preparation to obtain model-fit criteria before analysis to test multiple paths between variables and analyze different parameters. Depending on the number of variables and sample size, models may reach saturation. To minimize the limitations of SEM, we used a more parsimonious model considering the main variables related to the outcome and paying careful attention to data preparation.

Another study limitation is the use of the Nyvad criteria. Although validated and used in international studies, the visual-tactile method may not provide a accurate assessment of caries activity. To minimize possible errors, examiners received prior training, obtaining an acceptable level of inter- and intra-rater reliability.

Despite performing additional analyses including only children with at least 12 teeth, we were not able to completely remove the confounding effect of the number of erupted teeth as our sample included very young children (12–30 months), most of whom had few teeth. Further research involving children with a complete set of deciduous teeth may help gain a better understanding of the relationship between SGA and S-ECC; however, these studies may be more susceptible to other biases and confounders, such as memory bias, loss of teeth due to caries or other reasons, and longer exposure to other risk factors, hampering the measurement of disease incidence and producing estimates that point in the direction of non-association.

Another limitation is the use of the Kramer criterion, which is based on Canadian birthweight standards, for Brazilian children, potentially leading to an overestimation of SGA children. The Kramer criterion seeks to minimize methodological biases of other birthweight for gestational age curves^45^. To minimize this problem, we also analyzed the association between SGA and E-ECC using the INTERGROWTH-21st standard, which uses data from Brazilian children. It is also important to highlight that the lower incidence of SGA children using this standard observed by this study may be explained by the fact that this measure tends to underestimate SGA^46^.

Despite these limitations, one of the strengths of this study is the use of SEM. This method allows the interpretation of causal structures to estimate direct and indirect effects (mediation) and provides better confounder control, as well as adjusting for common causes. This model also allowed us to build a matrix of causal relationships organized hierarchically into distal, intermediate, and proximal causes based on the hypothesis and testing of the overall fit of the model.

Another strength is that we used data from a cohort study with a large sample carried out in a socioeconomically disadvantaged region, giving high statistical power. The fact that we performed a sensitivity analysis using two different criteria for SGA was another strong point. Furthermore, caries assessments that consider disease activity identify early lesions, avoiding underestimation of the incidence of S-ECC. This type of assessment is therefore more appropriate for studies involving young children and considering the time it takes for lesions to progress to cavities. In addition, the presence of S-ECC was also assessed based on the proportion of decayed tooth surfaces. This approach is more appropriate as it considers disease severity.

## Conclusions

The present study showed an association between SGA and S-ECC and that age and number of erupted teeth can influence the incidence of caries due to time of exposure to risk factors. SGA is therefore associated with S-ECC in this age group. The fact that SGA also acts as a protective factor against caries is probably explained by delayed tooth eruption and shorter exposure time to cariogenic agents.

### Supplementary Information

Supplementary Information.

## Data Availability

Due to the sensitive nature of the questions asked in this study, respondents were assured that the raw data would remain confidential and would not be shared. While the data is confidential, further information can be requested by contacting Professor Erika Bárbara Abreu Fonseca Thomaz (Department of Public Health, Federal University of Maranhão, Rua Barão de Itapari, 155, Centro, 65020-070, São Luís, Maranhão, Brazil or ebthomaz@gmail.com).
