# Supplementary figures and images for: Small for gestational age and early childhood caries: the BRISA cohort study
Source: Sci Rep. 2023 Sep 1;13:14343. doi: 10.1038/s41598-023-41411-y (PMC10474029; doi:10.1038/s41598-023-41411-y)

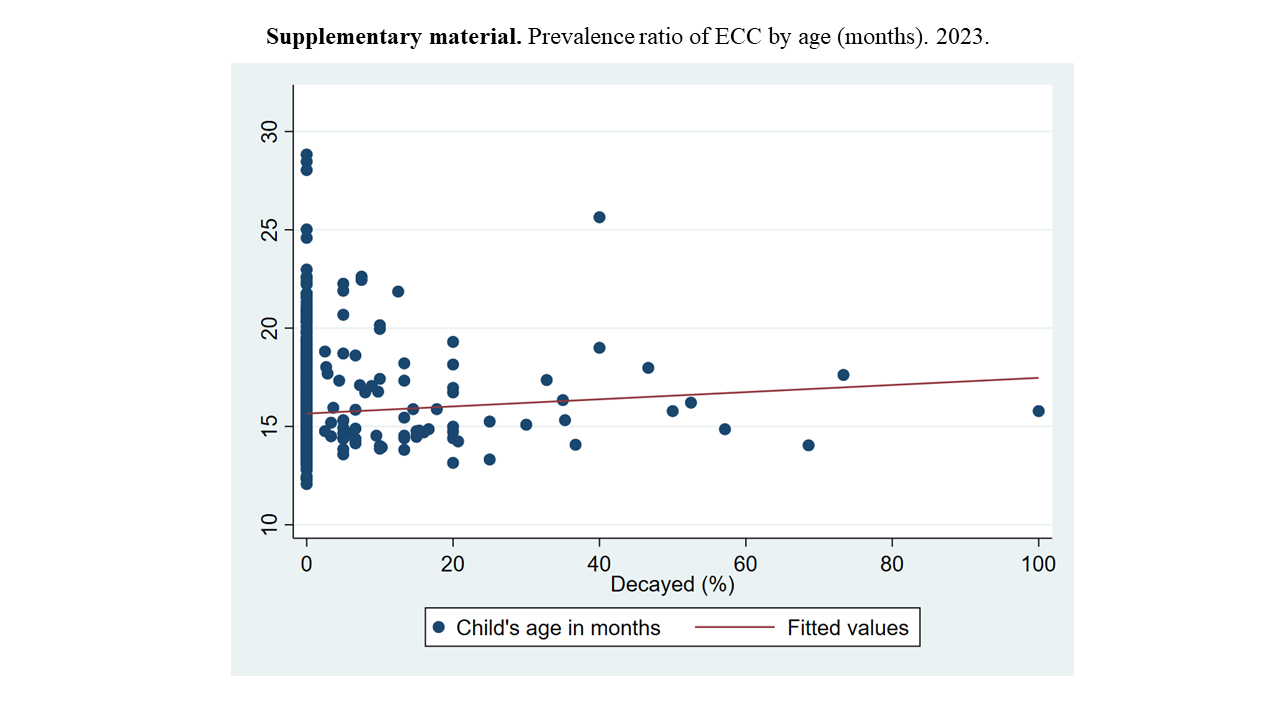

Supplement: Supplementary file 1 — Supplementary Information. [file 41598_2023_41411_MOESM1_ESM.tif]
